# Supplementary material for: Serial passaging affects stromal cell mechanosensitivity on hyaluronic acid hydrogels
Source: bioRxiv. 2023 Mar 20:2023.03.16.532853. Preprint. [Version 1] doi: 10.1101/2023.03.16.532853 (PMC10055097; doi:10.1101/2023.03.16.532853)
Supplement: Supplement 1 [file media-1.pdf]

Serial passaging affects stromal cell mechanosensitivity on hyaluronic acid hydrogels

Jenna L. Sumey[1], Abigail M. Harrell[2], Peyton C. Johnston[3], Steven R. Caliari[1,3]

[1]Department of Chemical Engineering, [2]Department of Chemistry, [3]Department of Biomedical Engineering, University of Virginia

Supporting Information

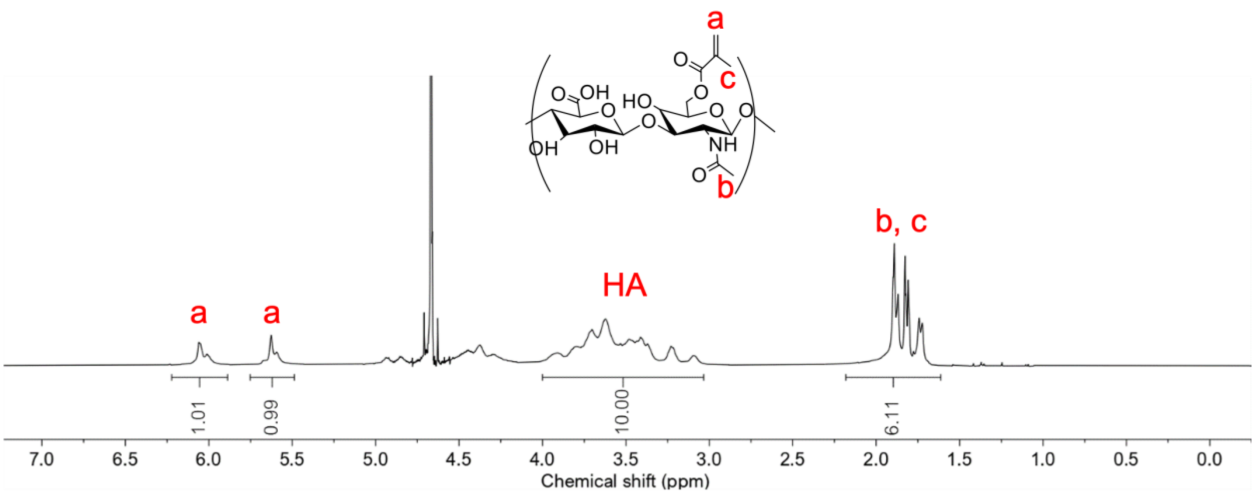

Figure S1. ^{1}H NMR spectrum of methacrylated hyaluronic acid (MeHA). The degree of modification was determined to be 100% as determined by the alkene peaks labeled a.

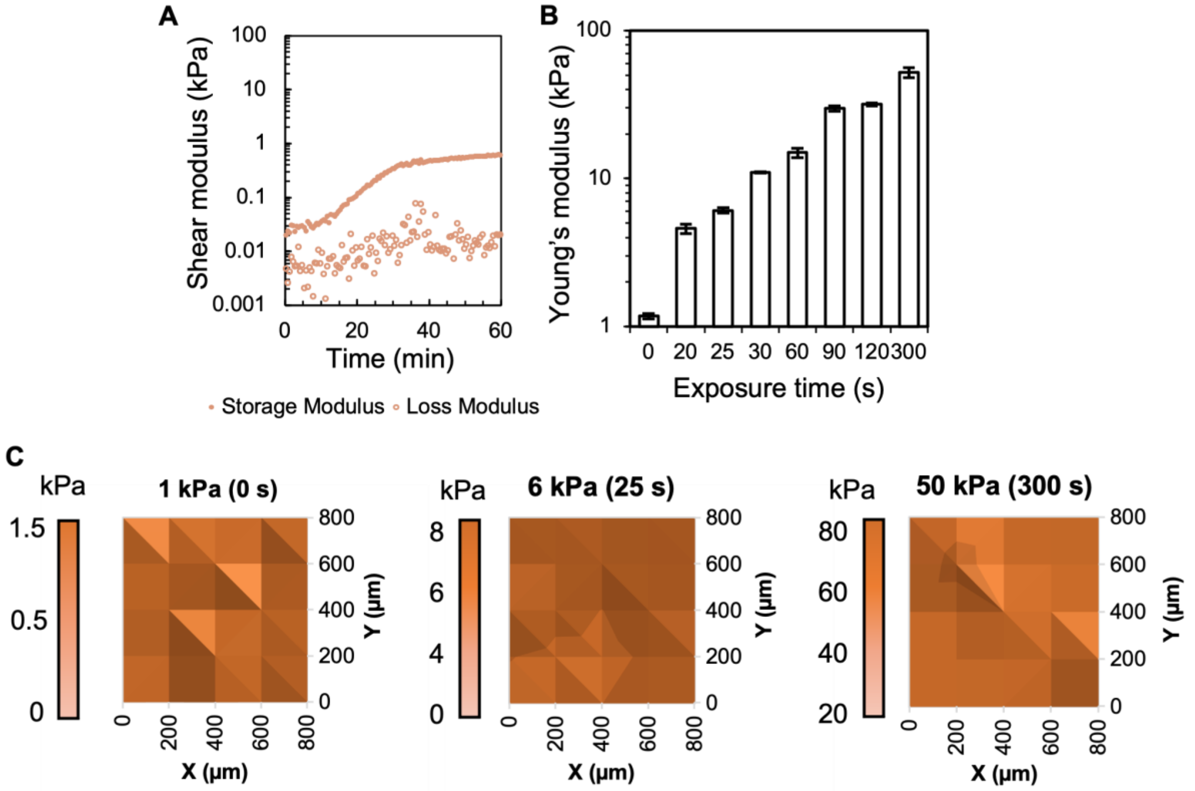

Figure S2. Mechanical characterization of MeHA hydrogels. **A**) *In situ* rheology of initial network formation. **B**) Nanoindentation of hydrogels following increasing lengths of blue light exposure in the presence of 2.2 mM LAP photoinitiator. **C**) Surface maps from nanoindentation to illustrate mechanical homogeneity across the hydrogels.

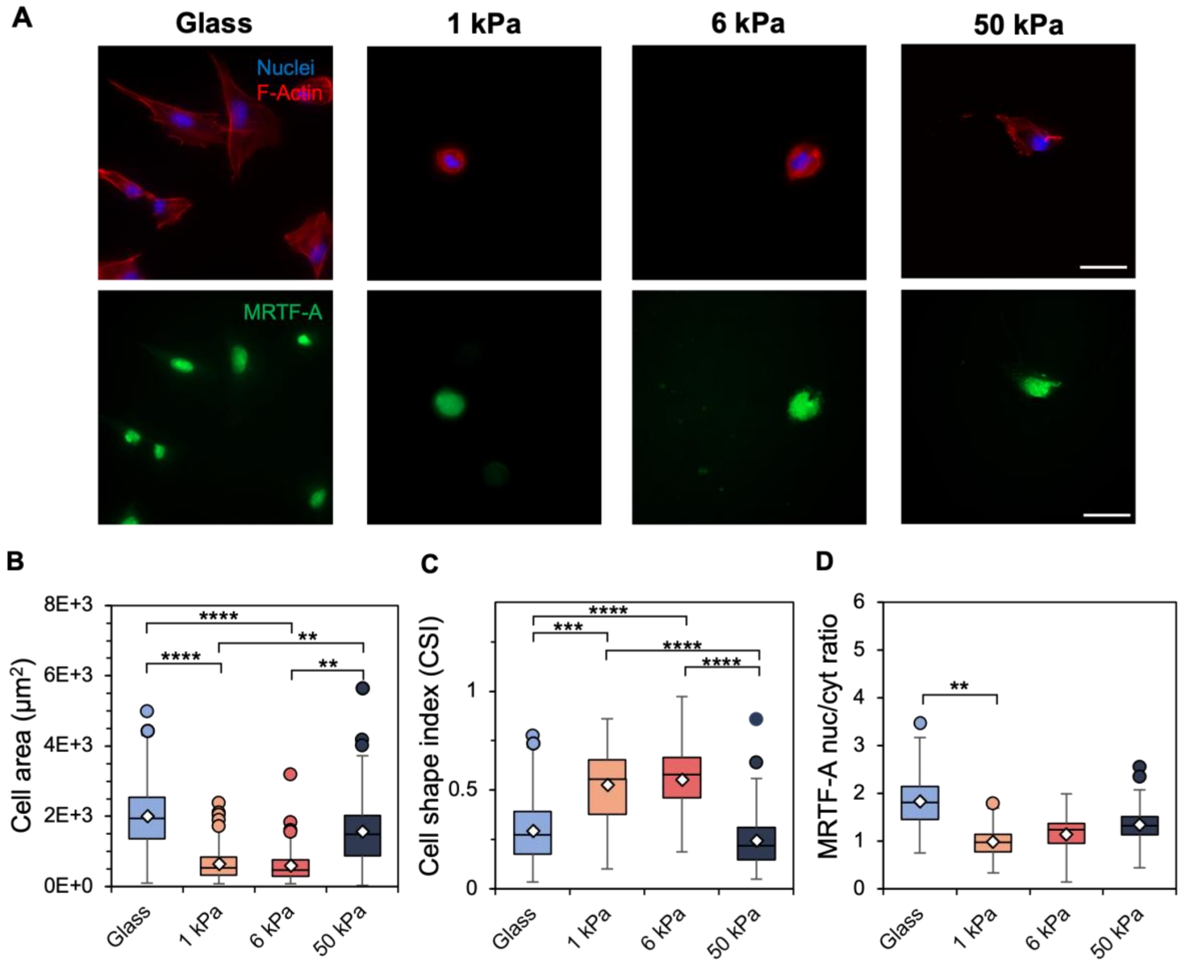

Figure S3. **A**) Representative images of passage 1 (P1) fibroblasts cultured on glass and 1, 6, and 50 kPa hydrogels for 4 days. Scale bars: 50 µm. Following 4 days of culture, quantification of **B**) cell spread area (µm^2), **C**) cell shape index, a measure of cell circularity, and **D**) MRTF-A nuclear-to-cytosol ratio was performed. *n* = 3 hydrogels per group. **** $P < 0.0001$, *** $P < 0.001$, ** $P < 0.01$.

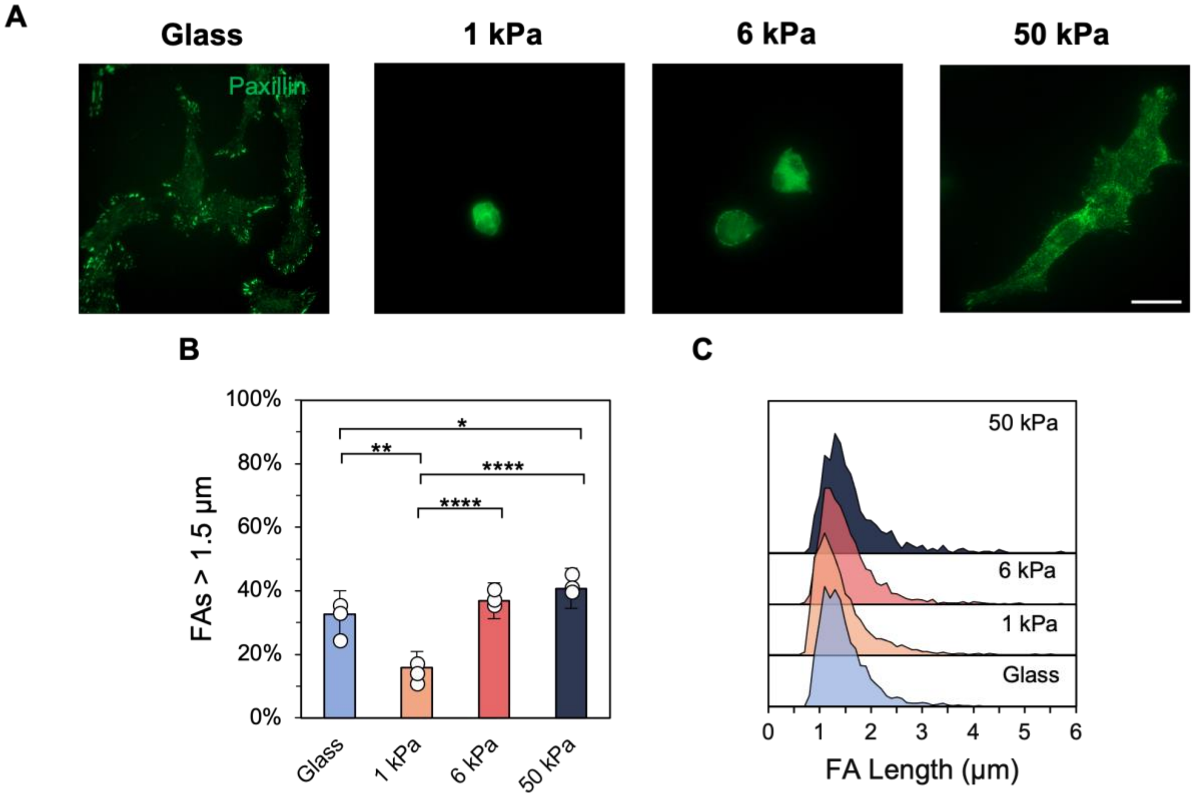

Figure S4. **A**) P1 fibroblasts were stained for paxillin to visualize focal adhesions, with quantification of **B**) focal adhesion lengths larger than 1.5 µm, a metric for mature adhesions, and **C**) ridgeline plots of the adhesion length distribution. Scale bar: 50 µm. $n = 3$ hydrogels per group. **** $P < 0.0001$, ** $P < 0.01$, * $P < 0.05$.

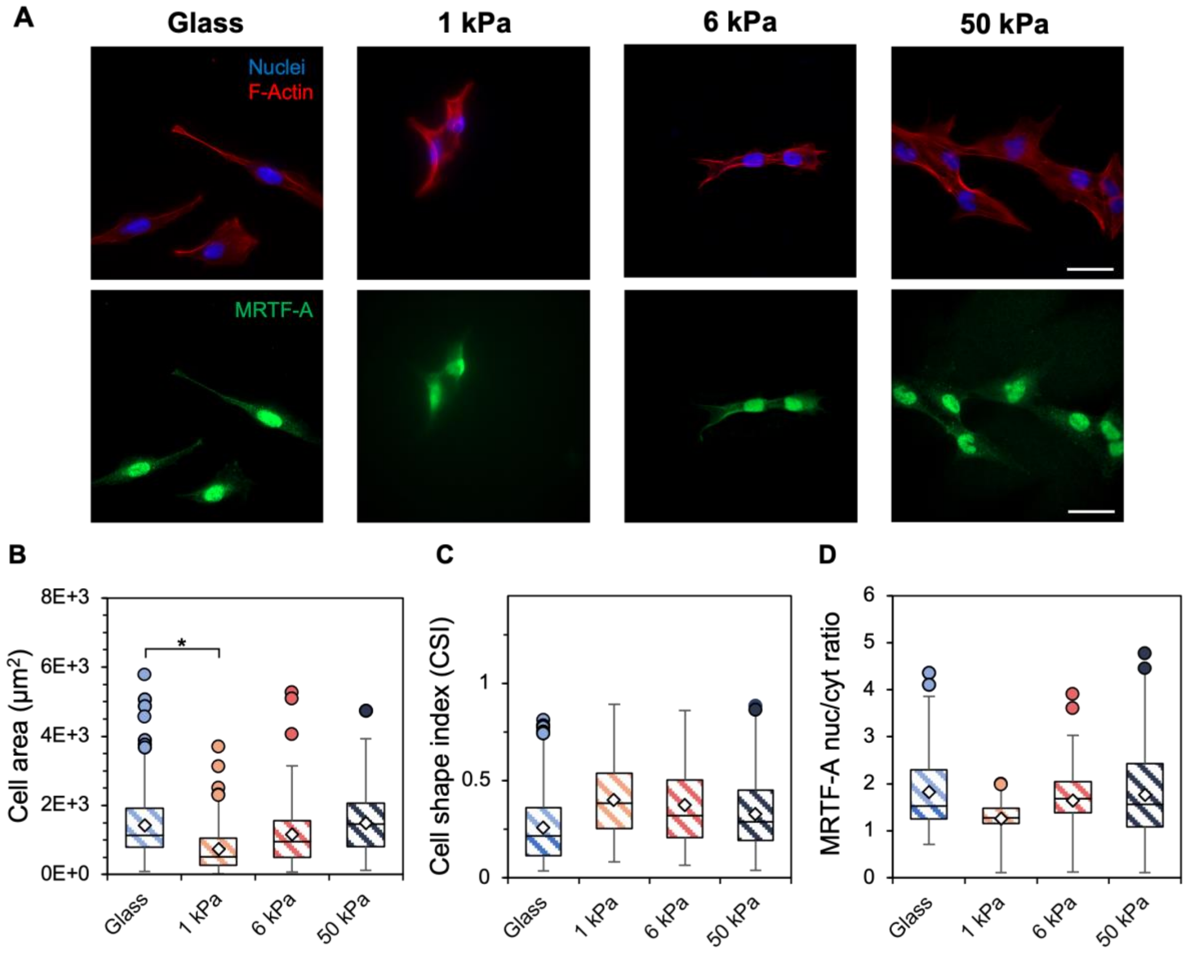

Figure S5. **A**) Representative images of passage 10 (P10) fibroblasts cultured on glass and 1, 6, and 50 kPa hydrogels for 4 days. Scale bars: 50 µm. Following 4 days of culture, quantification of **B**) cell spread area (µm^2), **C**) cell shape index, a measure of cell circularity, and **D**) MRTF-A nuclear-to-cytosol ratio was performed. n = 3 hydrogels per group. * $P < 0.05$.

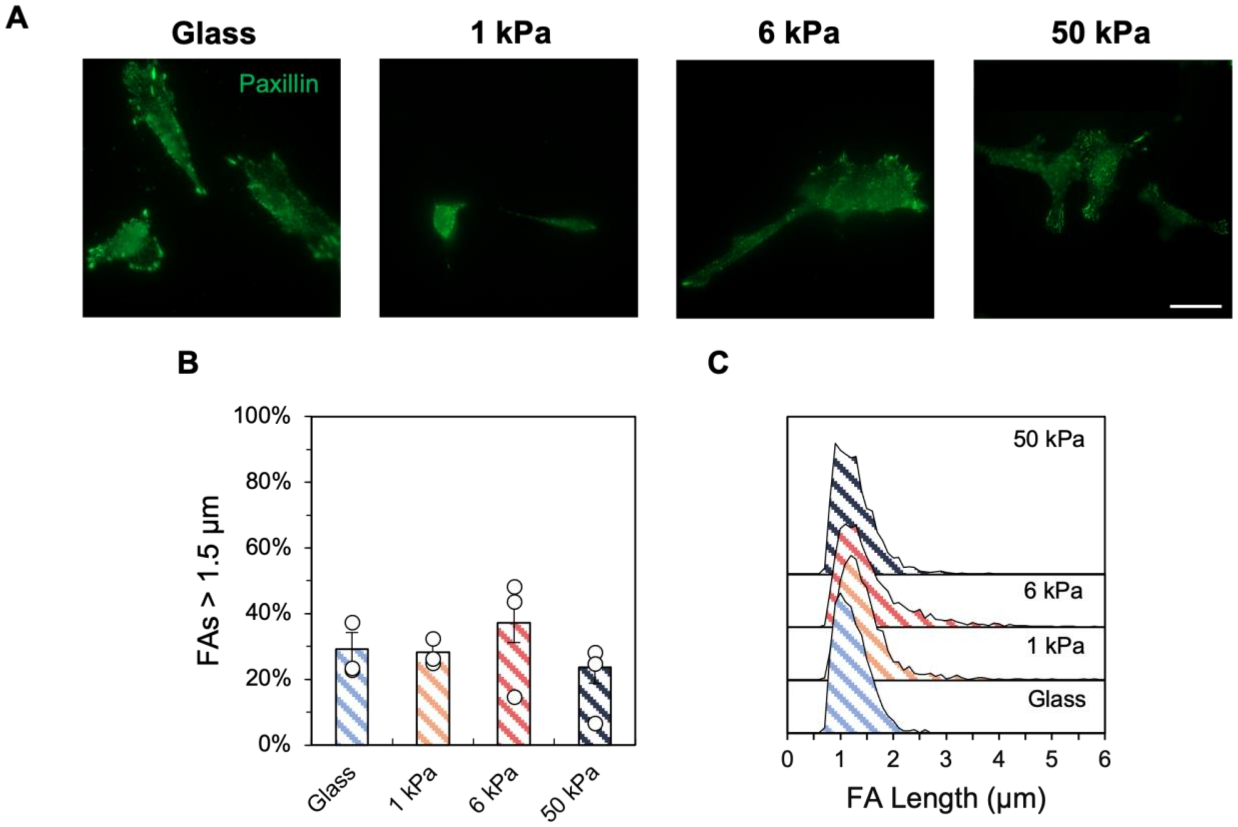

Figure S6. **A**) P10 fibroblasts were stained for paxillin to visualize focal adhesions, with quantification of **B**) focal adhesion lengths larger than 1.5 µm, a metric for mature adhesions, and **C**) ridgeline plots of the adhesion length distribution. Scale bar: 50 µm. *n* = 3 hydrogels per group. No statistically significant differences were observed.

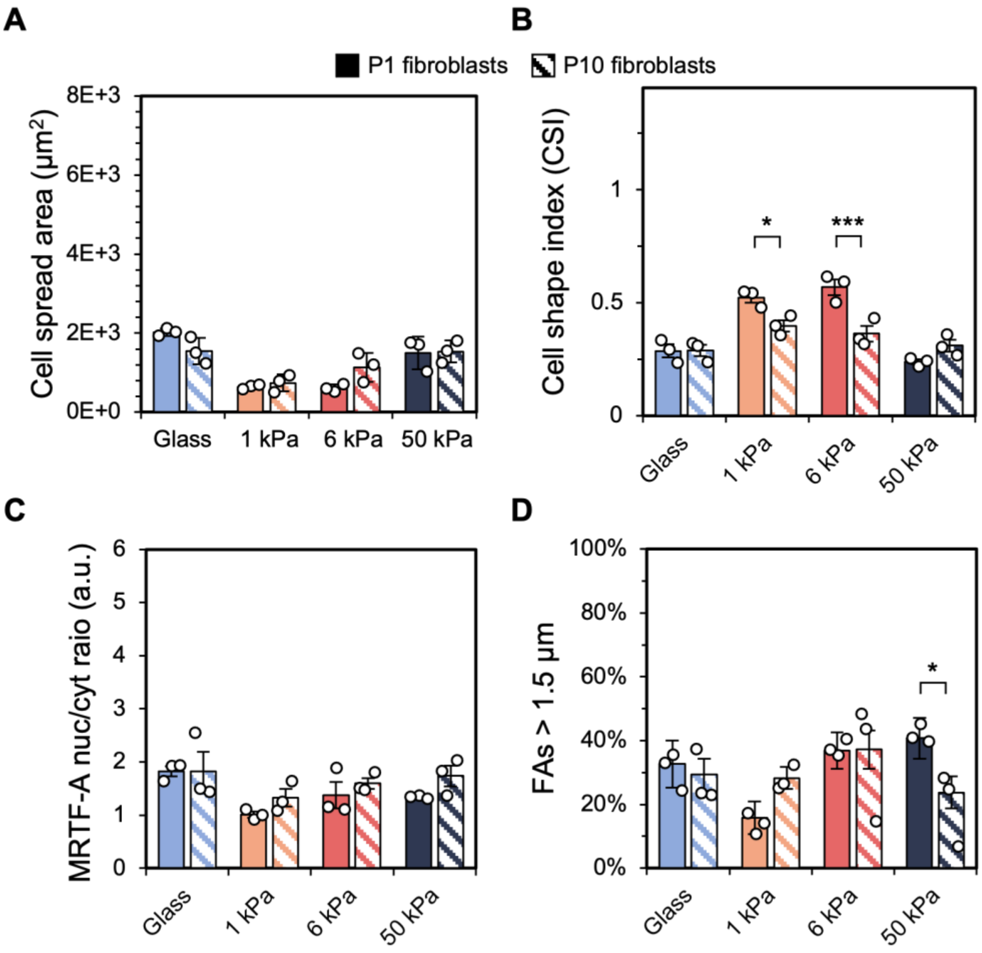

Figure S7. Comparison between P1 (solid bars) and P10 (striped bars) fibroblasts with respect to **A**) cell spread area, **B**) cell shape index, **C**) MRTF-A nuclear-to-cytosol ratio, and **D**) focal adhesion lengths larger than 1.5 μm following 4 days of hydrogel culture. Data are reported as the mean ± S.E.M. $n = 3$ hydrogels per group. * $P < 0.05$.
